# Supplementary material for: Humans disrupt access to prey for large African carnivores
Source: eLife. 2020 Nov 18;9:e60690. doi: 10.7554/eLife.60690 (PMC7673783; doi:10.7554/eLife.60690)
Supplement: Supplementary file 1. — Asterisks (*) indicate significant shifts in diel activity distributions due to human presence. Changes in nocturnality are depicted for species with significant increases (+) and decreases (-) in response to humans. Empty cells represent no significant changes. [file elife-60690-supp1.docx]

***Supplemental Information***

**Humans disrupt access to prey for large African carnivores**

Mills and Harris

**Table S1:** Species detections (using 30-minute quiet periods) during the camera survey and common diel period. Asterisks (*) indicate significant shifts in diel activity distributions due to human presence. Changes in nocturnality are depicted for species with significant increases (+) and decreases (-) in response to humans. Empty cells represent no significant changes.

| Species |  | Detections | Diel period^1^ | Significant diel shift | Change in nocturnality |
| --- | --- | --- | --- | --- | --- |
| **Apex Predators** | | **786** |  | ***** |  |
| Hyena | *Crocuta crocuta* | 628 | crepuscular | * |  |
| Leopard | *Panthera pardus* | 62 | nocturnal | * |  |
| Lion | *Panthera leo* | 96 | nocturnal |  |  |
| **Ungulates** | | **10,325** |  | ***** | **+** |
| Buffalo | *Syncerus caffer brachyceros* | 698 | cathemeral |  |  |
| Roan Antelope | *Hippotragus equinus koba* | 990 | diurnal | * |  |
| Hartebeest | *Alcelaphus buselaphus major* | 211 | diurnal |  |  |
| Waterbuck | *Kobus ellipsiprymnus defassa* | 161 | diurnal |  |  |
| Bushbuck | *Tragelaphus sylvaticus* | 2245 | cathemeral | * | + |
| Kob | *Kobus kob kob* | 847 | diurnal | * | - |
| Reedbuck | *Redunca redunca* | 1270 | cathemeral | * | + |
| Aardvark | *Orycteropus afer* | 261 | nocturnal | * | - |
| Warthog | *Phacochoerus africanus* | 1170 | diurnal | * | + |
| Duiker | *Sylvicapra grimmia* | 1488 | diurnal | * | + |
|  | *Cephalophus rufilatus* |  |  |  |  |
| Oribi | *Ourebia ourebi* | 984 | diurnal | * |  |

**^1^** Lamarque, F. (2004). *Les grands mammifères du complexe WAP*. CIRAD-ECOPAS, Montpellier.
