# Supplementary material for: Humans disrupt access to prey for large African carnivores
Source: eLife. 2020 Nov 18;9:e60690. doi: 10.7554/eLife.60690 (PMC7673783; doi:10.7554/eLife.60690)
Supplement: Supplementary file 2. — Detection (p) and occupancy (ψ) were modeled using the following covariates: CAM = camera type, SAV = percent savanna, YR = survey year, SITE = survey site, MGMT = management type (national park or hunting concession). [file elife-60690-supp2.docx]

***Supplemental Information***

**Humans disrupt access to prey for large African carnivores**

Mills and Harris

**Table S2**: Human occupancy model selection table of top models with ΔAICc < 2 derived from camera data collected over 3 survey seasons in the W-Arly-Pendjari complex, West Africa. Detection (*p*) and occupancy (𝜓) were modeled using the following covariates: CAM = camera type, SAV = percent savanna, YR = survey year, SITE = survey site, MGMT = management type (national park or hunting concession).

| Candidate Models for Human Occupancy | AICc | ΔAICc | AICc weight | χ^2^ Goodness-of-fit P-value | Occupancy Estimates | |
| --- | --- | --- | --- | --- | --- | --- |
|  |  |  |  |  | Mean | Var. |
| *p* (CAM + SAV + YR + SITE)  𝜓 (YR) | 826.73 | 0.00 | 0.47 | 0.327 | 0.543 | 0.160 |
| *p* (CAM + SAV + YR + SITE + TN)  𝜓 (YR) | 828.67 | 1.94 | 0.18 | 0.363 | 0.545 | 0.160 |
| *p* (CAM + SAV + YR + SITE)  𝜓 (YR + MGMT) | 828.70 | 1.97 | 0.18 | 0.359 | 0.540 | 0.166 |
| *p* (SAV + YR + SITE)  𝜓 (YR) | 828.73 | 2.00 | 0.17 | 0.353 | 0.548 | 0.153 |
