# Supplementary material for: Humans disrupt access to prey for large African carnivores
Source: eLife. 2020 Nov 18;9:e60690. doi: 10.7554/eLife.60690 (PMC7673783; doi:10.7554/eLife.60690)
Supplement: Supplementary file 3. — P-values are given for tests on species shifts using each threshold value. Sig. indicates the observed significance of shifts using the mean threshold value (0.54): + < 0.1, *<0.05, **<0.01, ***<0.001. The number of significant results (p-value<0.05) using different threshold values is given, for which three indicates significance using all thresholds and 0 indicates no significance for any threshold. [file elife-60690-supp3.docx]

***Supplemental Information***

**Humans disrupt access to prey for large African carnivores**

Mills and Harris

**Table S3**: Sensitivity analysis of species shifts in circular activity distributions, by adjusting the threshold value of human occupancy ± 0.1 from the mean. P-values are given for tests on species shifts using each threshold value. Sig. indicates the observed significance of shifts using the mean threshold value (0.54): + < 0.1, * < 0.05, ** < 0.01, *** < 0.001. The number of significant results (*p-*value < 0.05) using different threshold values is given, for which 3 indicates significance using all thresholds and 0 indicates no significance for any threshold.

| Species | Human occupancy threshold | | | Sig. | # sig. |
| --- | --- | --- | --- | --- | --- |
|  | 0.44 | 0.54 | 0.64 |  |  |
| **Apex predators** | 0.035 | 0.019 | 0.016 | * | 3 |
| Hyena | 0.012 | 0.014 | 0.005 | * | 3 |
| Leopard | 0.044 | 0.060 | 0.056 | + | 1 |
| Lion | 0.518 | 0.266 | 0.286 |  | 0 |
| **Ungulates** | 0 | 0 | 0 | *** | 3 |
| Aardvark | 0.001 | 0.012 | 0.017 | * | 3 |
| Buffalo | 0.451 | 0.406 | 0.238 |  | 0 |
| Bushbuck | 0 | 0 | 0 | *** | 3 |
| Duiker | 0.027 | 0.019 | 0.019 | * | 3 |
| Hartebeest | 0.363 | 0.229 | 0.233 |  | 0 |
| Kob | 0 | 0 | 0 | *** | 3 |
| Oribi | 0 | 0 | 0.001 | *** | 3 |
| Reedbuck | 0 | 0 | 0 | *** | 3 |
| Roan Antelope | 0.026 | 0.076 | 0.107 | + | 1 |
| Warthog | 0 | 0 | 0.001 | *** | 3 |
| Waterbuck | 0.053 | 0.118 | 0.098 |  | 0 |

**References:**

Mills, K.L., Harissou, Y., Gnoumou, I.T., Abdel-Nasseer, Y.I., Doamba, B. & Harris, N.C. (2020). Comparable space use by lions between hunting concessions and national parks in West Africa. *J. Appl. Ecol.*, 57, 975–984.
